# A retrospective population-based cohort study identifying target areas for prevention of acute lower respiratory infections in children

**DOI:** 10.1186/1471-2458-10-757

**Published:** 2010-12-07

**Authors:** Hannah C Moore, Nicholas de Klerk, Peter Richmond, Deborah Lehmann

**Affiliations:** 1 Telethon Institute for Child Health Research, Centre for Child Health Research, University of Western Australia, Perth, Australia; 2 School of Paediatrics and Child Health, University of Western Australia, Perth, Australia; 3 Princess Margaret Hospital for Children, Perth, Australia

## Abstract

**Background:**

Acute lower respiratory infections (ALRI) are a major cause of hospitalisation in young children. Many factors can lead to increased risk of ALRI in children and predispose a child to hospitalisation, but population attributable fractions for different risk factors and how these fractions differ between Indigenous and non-Indigenous children is unknown. This study investigates population attributable fractions of known infant and maternal risk factors for ALRI to inform prevention strategies that target high-risk groups or particular risk factors.

**Methods:**

A retrospective population-based data linkage study of 245,249 singleton births in Western Australia. Population attributable fractions of known maternal and infant risk factors for hospitalisation with ALRI between 1996 and 2005 were calculated using multiple logistic regression.

**Results:**

The overall ALRI hospitalisation rate was 16.1/1,000 person-years for non-Aboriginal children and 93.0/1,000 for Aboriginal children. Male gender, being born in autumn, gestational age <33 weeks, and multiple previous pregnancies were significant risk factors for ALRI in both Aboriginal and non-Aboriginal children. In non-Aboriginal children, maternal smoking during pregnancy accounted for 6.3% (95%CI: 5.0, 7.6) of the population attributable fraction for ALRI, being born in autumn accounted for 12.3% (10.8, 13.8), being born to a mother with three or more previous pregnancies accounted for 15.4% (14.1, 17.0) and delivery by elective caesarean accounted for 4.1% (2.8, 5.3). In Aboriginal children, being born to a mother with three or more previous pregnancies accounted for 16.5% (11.8, 20.9), but remote location at birth accounted for 11.7% (8.5, 14.8), maternal age <20 years accounted for 11.2% (7.8, 14.5), and being in the most disadvantaged socio-economic group accounted for 18.4% (-6.5, 37.4) of the population attributable fraction.

**Conclusions:**

The population attributable fractions estimated in this study should help in guiding public health interventions to prevent ALRI. A key risk factor for all children is maternal smoking during pregnancy, and multiple previous pregnancies and autumnal births are important high-risk groups. Specific key target areas are reducing elective caesareans in non-Aboriginal women and reducing teenage pregnancies and improving access to services and living conditions for the Aboriginal population.

## Background

Acute lower respiratory infections (ALRIs) are a leading cause of hospitalisation in young children, particularly in those under the age of 2 years [1]. Factors leading to an increased risk of ALRI in young children include foetal growth measures, male gender, number of children in the household, maternal education, maternal age, maternal smoking and asthma and low socio-economic status [2-8]. Foetal growth measures (short gestation and birthweight) are the most commonly investigated risk factors, but studies have shown discrepant results [2-4]. Additionally, studies investigating the factors associated with increased risk of ALRI in children have generally been conducted in small community settings giving results that may not be generalisable to the wider population [4,5,9], or have been conducted over a decade ago [6].

While several factors have been associated with ALRI, the clinical significance of each factor and the context of developing preventive measures at a population level has generally been overlooked. This can be overcome by assessing the population attributable fraction (PAF) which takes into account the level of the exposure in the population and estimates the proportion of the disease risk in a population that can be attributed to the causal effects of a risk factor or set of risk factors [10,11]. By estimating PAFs we can determine the proportion of disease that might be prevented if an exposure could be eliminated and hence help plan appropriate public health preventive measures [12]. Interactions between foetal growth measures and socio-economic status, maternal asthma and smoking and the modifying effect of some of these risk factors on others needs to be explored, but the attributable fraction of risk factors alone can provide the basis on which to develop targeted interventions to those most in need.

In industrialised countries, Indigenous populations, including Aboriginal Australians, suffer high rates of ALRI [1,13], and rates of pneumonia hospitalisations in those under the age of 2 years are 13.5 times higher in Aboriginal than in non-Aboriginal children [13]. Few studies have investigated risk factors for ALRI separately for Indigenous and non-Indigenous populations. Rather, studies have included ethnicity as a risk factor and report that Indigenous or minority groups have an increased risk of ALRI [14,15]. We know that in Western Australia the age and seasonal distribution of respiratory viruses differs between Aboriginal and non-Aboriginal children [16]. It is therefore reasonable to expect that the relative importance of infant and maternal risk factors for ALRI will differ between Aboriginal and non-Aboriginal children. Using the total population-based Western Australian Data Linkage System (WADLS) [17], we have sufficient power and accurate identification of Aboriginal status to investigate a substantial number of risk factors for severe ALRI at a population level over many years. Here we have used the WADLS to investigate risk factors for those children who have been admitted to hospital with ALRI on one or more occasion. In particular, we investigate whether the combined and individual PAFs of known infant and maternal risk factors for hospitalisation for ALRI at the antenatal and natal period vary between Aboriginal and non-Aboriginal children. We hypothesise that the PAF of individual risk factors is low.

## Methods

### Setting and data sources

Western Australia covers one-third of Australia, approximately 2.5 million square kilometres with a population of 2.2 million [18]. The WADLS encompasses systematic record linkage of numerous administrative health datasets [19]. Using the WADLS we extracted data on all singleton live births in Western Australia between 1996 and 2005 from the following datasets: Midwives' Notification System detailing pregnancy, labour and birth details, Birth and Death Register, and the Hospital Morbidity Database System providing details of all hospital admissions throughout Western Australia. The WADLS has 100% coverage of data for hospital admissions throughout the state and >99% coverage of births on the Midwives' Notification System with a record linkage success rate of >99%.

We limited our analysis to singleton births as multiple births are associated with more pregnancy complications compared to single gestations [20] and therefore are likely to have a different risk profile with respect to ALRI. We identified hospital admissions for ALRI using International Classification of Diseases diagnosis codes [21,22]. A Perl program was designed to forward map codes from the 9^th ^version to the 10^th ^version using available mapping tables [23]. We used the principal diagnosis code and 20 additional diagnosis codes to identify admissions for ALRI in the following categories: pneumonia (J12-J18, B59, B05.2, B37.1, B01.2), bronchiolitis (J21), influenza (J10-J11), whooping cough (A37), bronchitis (J20) and unspecified ALRI (J22). ALRI admissions within 14 days of a previous ALRI admission were classified as a single episode.

### Risk factors

The following maternal and infant risk factors were available from the WADLS for data analysis: maternal age (<20, 20-24, 25-29, 30-34 or ≥35 years), presence of smoking during pregnancy (yes/no), presence of maternal asthma during pregnancy (yes/no), gestational age (<33, 33-34, 35-36 or ≥37 weeks, to examine effects of prematurity), infant gender, number of previous pregnancies (0, 1, 2 or ≥3), mode of delivery (vaginal, instrumental, elective caesarean or emergency caesarean as recorded on the Midwives' Notification Form), and season of birth (summer, autumn, winter or spring). An elective caesarean is defined as a planned procedure prior to the onset of labour and before spontaneous rupture of membranes and without any procedure to induce labour. Proportion of optimal birthweight (POBW), a measure which takes into account gestational duration, foetal gender, maternal age, maternal height and parity [24] was used as a measure of gestational age-specific appropriateness of foetal growth, rather than birthweight alone. POBW was grouped into three categories (low <85%, normal 85-114% or high ≥115%). The Socio-Economic Index for Area (SEIFA) is comprised of several indices, the main index being the index for relative disadvantage which is derived from low income, low educational attainment, high unemployment and jobs in unskilled occupations [25]. This was used as a measure of disadvantage for each collection district (grouping of approximately 200 dwellings) in Australia. The collection district is the smallest unit available for population-based analyses. SEIFA scores are grouped into quantiles based on national statistics corresponding to the closest census year, either 1996 or 2001 [25,26]. The Accessibility/Remoteness Index of Australia was used as a specific measure of remoteness and access to services [27]. This index classifies the population into five categories (major cities, inner regional, outer regional, remote, or very remote) based on postcode of residence recorded at the time of birth. All datasets provided information concerning Aboriginal status and a child was identified as such if at least one record in one of the datasets recorded the child as Aboriginal. Approval for the study was sought and provided by the Princess Margaret Hospital for Children Ethics Committee and the Western Australian Aboriginal Health Information and Ethics Committee. Access to data from WADLS was approved by the Confidentiality of Health Information Committee and the Western Australian Data Linkage Branch.

### Statistical analysis

Person-time-at-risk was used to calculate age-specific incidence rates separately for Aboriginal and non-Aboriginal children for the following age groups: <1, 1-2, 3-5, 6-11, 12-23 months, 2-4 and 5-9 years. The proportion of children admitted at least once between 1996 and 2005 for pneumonia, bronchiolitis or influenza with each of the risk factors was first assessed to determine the direction of risk for each factor and to inform multivariate analysis. Multiple logistic regression was then used to generate separate models for Aboriginal and non-Aboriginal children with the outcome being at least one admission for ALRI before age 2 years (ie any admission versus no admission). Adjusted PAFs and a combined PAF were calculated using the *aflogit *command in Stata [28] where the combined PAF estimates the proportional amount by which disease risk would be reduced if all the risk factors were simultaneously eliminated from the population [10]. While non-modifiable factors cannot be eliminated, the combined PAF is useful to highlight how much of the disease risk is attributed to all the factors included in the model. Dummy variables for all risk factors were generated with the reference level for each factor being the category with the lowest risk as determined by the initial descriptive analysis. This was to ensure that the PAFs were derived from positive associations with the outcome. We report odds ratios (ORs) and 95% confidence intervals (CIs) from univariate analyses for each risk factor separately in Aboriginal and non-Aboriginal children adjusted only for year of birth and then adjusted ORs, PAF and 95%CIs from multivariate models including all the risk factors. All data cleaning was conducted in SPSS version 15.0 and analysis was conducted in Stata version 10.0.

## Results

Between 1996 and 2005, there were 26,106 episodes of ALRI identified in the birth cohort of 245,249 children, 7.1% (17,466) of whom identified as Aboriginal. The overall ALRI admission rate was 16.1/1,000 person-years for non-Aboriginal children and 93.0/1,000 person-years for Aboriginal children. Bronchiolitis accounted for 11,988 (45.9%) ALRI episodes (8,710 non-Aboriginal and 3,278 Aboriginal). Pneumonia was the next most common ALRI diagnosis, accounting for 29.6% of all episodes (5,181 non-Aboriginal and 2,546 Aboriginal) and influenza accounted for 4.7%.

The highest hospitalisation rate for bronchiolitis in non-Aboriginal children was in those aged 1-2 months and in Aboriginal children aged 3-5 months, for influenza in children aged 1-5 months and for pneumonia, the highest hospitalisation rate in non-Aboriginal children was in those aged 12-23 months and in Aboriginal children aged 6-11 months (Table 1). Generally, ALRI admission rates were lower in children aged 2 years or more compared with rates in younger children (Table 1). The biggest relative disparity in admission rates between Aboriginal and non-Aboriginal children was for pneumonia; for example, in children aged 3-5 months the hospitalisation rate for pneumonia was 15 times higher in Aboriginal than in non-Aboriginal children.

**Table 1:** Frequency of hospitalisations by ALRI diagnosis and age group in Aboriginal and non-Aboriginal children

ALRI diagnosis	Age group	Number of hospitalisations (Rate^a^)			
		
		Aboriginal		Non-Aboriginal	
Whooping cough	<1 month	6	(4.2)	25	(1.3)
	1-2 months	36	(12.7)	101	(2.7)
	3-5 months	42	(10.1)	62	(1.1)
	6-11 months	13	(1.6)	26	(0.2)
	12-23 months	6	(0.4)	23	(0.1)
	2-4 years	0	-	10	(0.0)
	5-9 years	1	(0.05)	6	(0.02)
Pneumonia	<1 month	42	(29.1)	136	(7.2)
	1-2 months	118	(41.6)	133	(3.6)
	3-5 months	241	(57.9)	212	(3.9)
	6-11 months	570	(71.7)	629	(6.0)
	12-23 months	740	(51.0)	1,614	(8.4)
	2-4 years	711	(21.4)	1,937	(4.4)
	5-9 years	124	(6.0)	520	(1.8)
Bronchiolitis	<1 month	119	(82.4)	546	(28.9)
	1-2 months	579	(204.0)	1,958	(52.6)
	3-5 months	923	(222.1)	2,221	(40.7)
	6-11 months	1,090	(137.2)	2,510	(24.0)
	12-23 months	467	(32.2)	1,172	(6.1)
	2-4 years	97	(2.9)	281	(0.6)
	5-9 years	3	(0.1)	22	(0.1)
Influenza	<1 month	4	(2.8)	23	(1.2)
	1-2 months	20	(7.0)	72	(1.9)
	3-5 months	29	(7.0)	101	(1.9)
	6-11 months	43	(5.4)	179	(1.7)
	12-23 months	41	(2.8)	270	(1.4)
	2-4 years	42	(1.3)	321	(0.7)
	5-9 years	9	(0.4)	80	(0.3)
Bronchitis	<1 month	3	(2.1)	2	(0.1)
	1-2 months	20	(7.0)	24	(0.6)
	3-5 months	46	(11.1)	52	(1.0)
	6-11 months	69	(8.7)	107	(1.0)
	12-23 months	88	(6.1)	147	(0.8)
	2-4 years	69	(2.1)	135	(0.3)
	5-9 years	11	(0.5)	33	(0.1)
Unspecified ALRI	<1 month	15	(10.4)	35	(1.9)
	1-2 months	15	(20.4)	55	(1.5)
	3-5 months	119	(28.6)	88	(1.6)
	6-11 months	319	(40.1)	309	(3.0)
	12-23 months	480	(33.0)	803	(4.2)
	2-4 years	386	(11.7)	1,008	(2.3)
	5-9 years	65	(3.2)	254	(0.9)
Total ALRI	<1 month	189	(131.0)	767	(40.6)
	1-2 months	831	(292.7)	2,343	(62.9)
	3-5 months	1,400	(336.8)	2,736	(50.2)
	6-11 months	2,104	(264.8)	3,760	(36.0)
	12-23 months	1,822	(125.5)	4,029	(21.0)
	2-4 years	1,305	(39.4)	3,692	(8.4)
	5-9 years	213	(10.4)	915	(3.2)

^a^Rate per 1000 child-years at risk

One in four (25.6%) Aboriginal children were hospitalised at least once for ALRI compared with one in 15 (6.5%) non-Aboriginal children. The proportions of children admitted at least once for each level of the risk factors considered, were distributed similarly for pneumonia, bronchiolitis and influenza (see Additional file 1). Therefore, male gender, POBW <85%, gestational age <33 weeks, ≥3 previous pregnancies, being born in autumn or by caesarean section, maternal age <20 years, maternal smoking and asthma during pregnancy, most disadvantaged families or those residing in outer regional or remote areas with moderate to low access to services were identified as those groups with the highest proportion of children hospitalised for ALRI before age 2 years (see Additional file 1). As the risk factors were similar for pneumonia, bronchiolitis and influenza, logistic regression models were conducted using the outcome of ALRI rather than individual diagnostic categories of ALRI.

Logistic regression models calculating PAFs were restricted to ALRI episodes before age 2 years as the majority of ALRI episodes occurred in this age group. As no differences with regard to patterns of risk between pneumonia, bronchiolitis and influenza were observed, logistic regression models were generated with the outcome of any ALRI diagnosis.

In non-Aboriginal children the strongest association was for gestational age where for very preterm children (gestational age <33 weeks), the odds of an ALRI admission was 5 times higher compared with children born at ≥37 weeks gestation, independent of other risk factors (adjusted OR 4.70, 95% CI: 4.08, 5.41) (Table 2). In the adjusted analysis there was a 33% increase in the odds of ALRI admission if the mother smoked during pregnancy and a 47% increase if the mother had asthma during pregnancy (Table 2). There was a positive association between younger maternal age and risk of ALRI admission. The highest odds of ALRI was in children of teenage mothers (adjusted OR 2.60, 95% CI: 2.3, 2.94) compared to children of mothers aged 35 years or more. The combined PAF for non-Aboriginal children was 88.3% (95% CI: 84.3, 91.3), indicating that the factors included in the model accounted for most of the risk of hospitalisation. Adjusting for all other risk factors, the factors with the highest PAFs were male gender (16%), being born to a mother who already had three or more pregnancies (15%) and being born in autumn (months March to May, 12%). Maternal smoking during pregnancy accounted for 6% of the PAF, maternal asthma during pregnancy accounted for 5% and elective caesarean deliveries accounted for 4% (Table 2).

**Table 2:** Odds ratios and population attributable fractions for ALRI hospitalisation before age 2 years in non-Aboriginal children

Risk factor	**Univariate**^a^		Adjusted		Adjusted	
	
	OR	95% CI	OR	95% CI	PAF %	95% CI
Gender						
Female	Reference					
Male	1.39	1.34, 1.45	1.40	1.34, 1.47	16.0	13.8, 18.1
Gestational age						
≥37 weeks	Reference					
35-36 weeks	1.87	1.74, 2.02	1.70	1.54, 1.87	2.6	2.0, 3.1
33-34 weeks	2.18	1.91, 2.49	2.04	1.73, 2.42	1.0	0.7, 1.3
<33 weeks	4.84	4.35, 5.38	4.70	4.08, 5.41	2.7	2.3, 3.1
Percent optimal birthweight						
Low <85%	1.37	1.29, 1.46	1.14	1.06, 1.22	1.5	0.6, 2.3
Normal 85-114%	Reference					
High ≥115%	1.02	0.96, 1.09	1.02	0.95, 1.11	0.3	-0.5, 1.0
Number of previous pregnancies						
0	Reference					
1	1.47	1.40, 1.56	1.63	1.52, 1.74	11.3	9.7, 12.8
2	1.73	1.64, 1.84	2.00	1.86, 2.17	10.6	9.4, 11.8
≥3	2.12	2.00, 2.24	2.47	2.29, 2.66	15.4	14.1, 17.0
Season of birth						
Spring	Reference					
Summer	1.23	1.16, 1.30	1.24	1.16, 1.33	4.2	2.8, 5.6
Autumn	1.64	1.56, 1.73	1.72	1.61, 1.83	12.3	10.8, 13.8
Winter	1.39	1.31, 1.47	1.41	1.32, 1.51	7.0	5.6, 8.4
Mode of delivery						
Vaginal	1.40	1.31, 1.49	1.04	0.96, 1.13	2.2	-2.2, 6.5
Instrumental	Reference					
Elective caesarean	1.48	1.38, 1.60	1.34	1.22, 1.48	4.1	2.8, 5.3
Emergency caesarean	1.52	1.41, 1.65	1.20	1.09, 1.33	2.0	0.9, 3.1
Maternal smoking during pregnancy						
No	Reference					
Yes	1.79	1.70, 1.89	1.33	1.26, 1.41	6.3	5.0, 7.6
Maternal asthma during pregnancy						
No	Reference					
Yes	1.64	1.55, 1.74	1.47	1.37, 1.57	4.6	3.7, 5.5
Maternal age (years)						
≥35 years	Reference					
30-34 years	1.10	1.04, 1.18	1.21	1.12, 1.31	4.6	2.8, 6.4
25-29 years	1.31	1.23, 1.39	1.52	1.41, 1.65	10.2	8.4, 12.0
20-24 years	1.73	1.62, 1.84	1.97	1.80, 2.15	9.6	8.4, 10.8
<20 years	1.97	1.80, 2.15	2.60	2.30, 2.94	3.8	3.2, 4.4
SEIFA Index of Disadvantage^b^						
91-100%	Reference					
76-90%	1.23	1.11, 1.36	1.12	0.99, 1.25	1.2	-0.1, 2.5
26-75%	1.47	1.35, 1.61	1.10	0.99, 1.22	4.2	-0.2, 8.5
11-25%	1.92	1.74, 2.11	1.28	1.14, 1.43	4.3	2.4, 6.1
0-10%	2.23	2.01, 2.47	1.33	1.17, 1.50	2.8	1.6, 3.9
Accessibility/Remoteness Index of Australia						
Very remote	Reference					
Remote	1.47	1.16, 1.87	1.36	1.03, 1.81	1.2	0.2, 2.1
Outer regional	1.89	1.51, 2.37	1.62	1.25, 2.12	4.6	2.5, 6.8
Inner regional	1.31	1.04,1.64	1.05	0.81, 1.38	0.5	-2.1, 3.1
Major cities	1.23	0.99, 1.53	1.14	0.88, 1.48	8.4	-8.1, 22.4

^a^all adjusted for birth year

^b ^91-100% is least disadvantaged and 0-10% is most disadvantaged.

In Aboriginal children the largest association with ALRI admission was also with gestational age, independent of other risk factors; in this case very preterm children had an OR of 3.18 (Table 3). Similar to non-Aboriginal children, children of teenage mothers had the highest odds of ALRI compared to older mothers. Although the combined PAF for Aboriginal children was slightly higher than for non-Aboriginal children at 91.3% (95% CI: 76.0, 96.9), the individual PAFs were lower for several factors. The most disadvantaged children, with a SEIFA score in the 0-10% quantile, and those in very remote locations with poor access to services accounted for the highest PAFs for ALRI admission (18% for most disadvantaged and 12% for those in very remote locations) (Table 3). Similar to non-Aboriginal children, being of male gender accounted for 13% and being born to a mother with three or more previous pregnancies accounted for 17%. Adjusting for all other risk factors, maternal smoking during pregnancy accounted for 5% of the PAF and being born to a teenage mother accounted for 11% (Table 3). The results were similar when the outcome was restricted to admission for ALRI before age 6 months in both Aboriginal and non-Aboriginal children (data not shown).

**Table 3:** Odds ratios and population attributable fractions for ALRI hospitalisation before age 2 years in Aboriginal children

Risk factor	**Univariate**^a^		Adjusted		Adjusted	
	
	OR	95% CI	OR	95% CI	PAF %	95% CI
Gender						
Female	Reference					
Male	1.35	1.26, 1.45	1.42	1.28, 1.58	13.3	9.4, 17.1
Gestational age						
≥37 weeks	Reference					
35-36 weeks	1.44	1.26, 1.64	1.39	1.15, 1.69	1.8	0.6, 2.8
33-34 weeks	1.86	1.51, 2.29	1.71	1.27, 2.30	1.1	0.4, 1.8
<33 weeks	2.79	2.31, 3.35	3.18	2.42, 4.16	2.9	2.1, 3.7
Percent optimal birthweight						
Low <85%	1.65	1.39, 1.96	1.43	1.15, 1.78	5.8	2.3, 9.1
Normal 85-114%	1.18	1.01, 1.38	1.15	0.94, 1.40	6.8	-3.3, 15.9
High ≥115%	Reference					
Number of previous pregnancies						
0	Reference					
1	0.97	0.86, 1.08	1.03	0.87, 1.23	0.5	-1.9, 2.8
2	1.14	1.01, 1.28	1.39	1.15, 1.67	3.9	1.7, 6.1
≥3	1.28	1.16, 1.41	1.82	1.52, 2.19	16.5	11.8, 20.9
Season of birth						
Spring	Reference					
Summer	1.22	1.10, 1.35	1.22	1.05, 1.42	3.5	0.9, 6.1
Autumn	1.38	1.25, 1.53	1.46	1.27, 1.69	7.2	4.5, 9.9
Winter	1.25	1.12, 1.38	1.25	1.08, 1.45	3.9	1.3, 6.4
Mode of delivery						
Vaginal	Reference					
Instrumental	0.85	0.73, 0.98	1.23	0.99, 1.52	0.9	-0.1, 1.9
Elective caesarean	0.92	0.80, 1.05	1.04	0.85, 1.26	0.2	-0.9, 1.3
Emergency caesarean	1.09	0.98, 1.05	1.16	0.98, 1.37	1.3	-0.2, 2.7
Maternal smoking during pregnancy						
No	Reference					
Yes	1.33	1.23, 1.44	1.14	1.03, 1.27	5.1	1.1, 8.9
Maternal asthma during pregnancy						
No	Reference					
Yes	1.00	0.88, 1.14	1.05	0.89, 1.24	0.4	-0.9, 1.8
Maternal age (years)						
≥35 years	Reference					
30-34 years	1.09	0.92, 1.31	1.17	0.91, 1.51	1.5	-0.9, 3.8
25-29 years	1.21	1.03, 1.43	1.36	1.07, 1.73	5.4	1.3, 9.3
20-24 years	1.19	1.01, 1.41	1.55	1.21, 1.98	9.1	4.3, 13.6
<20 years	1.31	1.11, 1.55	2.17	1.66, 2.85	11.2	7.8, 14.5
SEIFA Index of Disadvantage^b^						
91-100%	Reference					
76-90%	0.74	0.35, 1.57	1.20	0.42, 3.38	0.2	-0.9, 1.3
26-75%	1.21	0.61, 2.39	1.70	0.65, 4.44	9.2	-5.8, 22.0
11-25%	1.37	0.69, 2.71	1.73	0.66, 4.51	9.3	-5.4, 22.0
0-10%	1.67	0.84, 3.30	1.94	0.75, 5.05	18.4	-6.5, 37.4
Accessibility/Remoteness Index of Australia						
Very remote	1.93	1.62, 2.30	2.09	1.68, 2.61	11.7	8.5, 14.8
Remote	1.15	0.96, 1.39	1.20	0.95, 1.52	2.0	-0.5, 4.4
Outer regional	1.36	1.14, 1.64	1.46	1.16, 1.84	4.3	1.7, 6.8
Inner regional	Reference					
Major cities	1.02	0.86, 1.21	1.08	0.87, 1.33	2.0	-3.7, 7.4

^a^all adjusted for birth year

^b ^91-100% is least disadvantaged and 0-10% is most disadvantaged.

## Discussion

Using total population-based data over 10 years and separating analyses for Aboriginal and non-Aboriginal children, we have shown that while many factors are associated with an increased risk of ALRI and the factors investigated contribute to 88-91% of the combined PAF for ALRI, the PAFs of individual risk factors are low. The key factors with notable PAFs are gender, season of birth, number of previous pregnancies, mode of delivery, maternal age and socio-economic status. The greatest use of PAFs is to highlight modifiable risk factors, predicting how much disease can be averted with their elimination [10] and then to direct concerted efforts to modifiable factors with the largest PAFs. Not all risk factors we have presented here are amenable to intervention or are even modifiable, but our analysis has highlighted differences and similarities in the level of importance of risk factors for ALRI in Aboriginal and non-Aboriginal singleton children and we highlight the areas that need to be targeted for ALRI prevention in these populations.

Similar to a retrospective cohort study in the United States of America [29], we found a strong association between seasonality of births and risk of ALRI with the highest risk in autumn-born children who were then aged 1-5 months in winter, the time when RSV is circulating [16] and infants are at the highest risk of ALRI, especially bronchiolitis [9]. This would suggest that, in order to reduce cost of RSV immunoprophylaxis with monoclonal antibody palivizumab which is recommended for high risk children [30], it might be better to target children based on their month of birth rather than on the timing of the RSV season alone. The relationship between number of previous pregnancies and risk of ALRI for Aboriginal and non-Aboriginal children could be seen as a proxy for crowding, where the highest risk of ALRI is in a child born to a mother who has previously had three or more pregnancies, although we acknowledge the outcome of these previous pregnancies is unknown. However, the likelihood of these families having a child of preschool age in the house is high, representing conditions favouring transmission of respiratory pathogens [31]. The increased risk with multiple number of pregnancies has also been reported in another Australian study with a combined analysis of Aboriginal and non-Aboriginal children [32].

Maternal smoking during pregnancy is an independent risk factor for ALRI and increases in risk in the order of 19-29% have been found in mothers who smoked during pregnancy [5,33,34]. We add to this evidence and report a 33% increase in odds for non-Aboriginal children and a 14% increase in odds for Aboriginal children; however, few other studies have used PAFs to compare to our estimates. In our study, in the presence of other factors, 6% of ALRI in non-Aboriginal children and 5% in Aboriginal children could be prevented if maternal smoking was eliminated. This is lower than a study conducted in an Indigenous population of Greenland that found a PAF of 47%, but this related to exposure to passive smoking around the time after birth and risk of ALRI in a community setting [9]. However, parental smoking should continue to be a priority for public health intervention as it is a modifiable risk factor. Gestational age has previously been identified as an important risk factor for ALRI [3,4]. Even though the odds of ALRI were almost 5-fold for non-Aboriginal and 3-fold for Aboriginal very preterm infants in our study, the PAF was only 3%.

We report differences in importance of various risk factors between Aboriginal and non-Aboriginal children indicating that different public health interventions need to be designed and implemented accordingly. For non-Aboriginal children, results suggest that 4% of ALRI could be prevented if there were no elective caesarean sections and lowest risk was in mothers who had an instrumental delivery, if the association is causal. This association with elective caesareans has been reported previously concentrating on neonatal respiratory morbidity [35], but the mechanisms underlying this association remain unclear and further studies are needed to understand this relationship. Similarly, maternal asthma was a significant risk factor in non-Aboriginal children but not in Aboriginal children. Maternal asthma has been found to be a more important risk factor for ALRI than smoking [36], but we found a similar PAF of maternal smoking and maternal asthma in pregnancy in non-Aboriginal children and maternal smoking is more amenable to intervention than maternal asthma.

There was an inverse relationship with maternal age with the highest risk of ALRI in children of teenage mothers, a finding that has also been reported previously [5]. This was especially in Aboriginal children in whom 11% of ALRI could be prevented if the association is causal and if there were no births to teenage mothers who represented almost a quarter of all Aboriginal mothers. More awareness is needed regarding the risks of teenage pregnancies and efforts to reduce the teenage pregnancy rate in the Aboriginal population need to be enhanced. Also, for Aboriginal children, the most disadvantaged socio-economic groups and those located in the very remote regions accounted for the highest PAFs. These results suggest that if living conditions and access to services were improved, a substantial proportion of ALRI hospitalisations could be prevented in this population and this would have a higher impact than prevention of smoking in pregnancy. However for general living conditions to improve in the Aboriginal population a multifaceted approach involving infrastructure such as housing and management [37], children's education and training of healthcare providers at the state government and local community level is needed.

While migration out of Western Australia for children aged less than four years is small [38], we are unable to estimate the proportion of individuals that moved around the state from their area of birth. This is due to privacy and confidentiality restrictions associated with obtaining data from the WADLS. Therefore socio-economic status and the accessibility/remoteness index may have changed between birth and time of hospitalisation, but we believe this to have little impact on our results. There are other potential risk factors that were not available in our current dataset such as paternal smoking, whether assisted reproduction was used, presence and duration of breastfeeding, immunisation status and child care attendance. Instead our emphasis has been on maternal and infant factors in the antenatal and natal period. We are currently unable to assess the impact of vaccines due to the non-availability of data, although we are planning to link individual immunisation data to the WADLS to address this issue at an individual level. Another limitation of our study is the quality of the data on risk factors especially in regards to maternal smoking and asthma. Recording of these measures on the Midwives' Notification System only commenced in 1997 and has not been validated. One study alluded that several other measures on the Midwives' Notification System, including mode of delivery, have high specificity but low sensitivity (E Blair, personal communication 2009). Therefore we may be underestimating the relationship between some of these factors and risk of ALRI and therefore underestimating the PAF. In multiple risk factor analysis there is the inherent problem of colinearity between factors. This has been noted previously in one study where gestational age was not an independent risk factor as it was related to so many other factors [14] and another where the presence of maternal asthma modified the risk of preterm delivery [39].

## Conclusion

This is one of the few studies to report PAFs for ALRI and the first study to assess PAFs separately for Aboriginal and non-Aboriginal populations. The WADLS captures information on >99% of births in WA with accurate identification of Aboriginal status and this has given us the opportunity to conduct meaningful analyses with sufficient power. We have highlighted areas that require a more targeted approach for intervention, those factors that need to be targeted separately in Aboriginal and non-Aboriginal children and those factors that are not modifiable but highlight susceptible subgroups that need to have increased awareness of the higher risk of ALRI. As there are many factors that span lifestyle, environmental and social aspects leading to ALRI, a multifaceted approach is needed to move towards prevention. In the first instance, increased RSV immunoprophylaxis measures for autumn-born babies with other risk factors, and interventions targeting maternal smoking during pregnancy need attention and further analysis is needed to understand the associations with teenage pregnancies in Aboriginal women and elective caesareans in non-Aboriginal women. Infants in the first six months of life are at a high risk of ALRI and efforts such as education around infection control measures and hygiene including hand-washing need to be reinforced. Finally, PAFs are useful in determining the areas that need to be targeted for prevention, especially where causality can be assumed, and they should be reported more widely.

## Competing interests

The authors declare that they have no competing interests.

## Authors' contributions

All authors developed the study design. HM cleaned and analysed the data and drafted the manuscript. NdK provided expert statistical advice. PR provided expert clinical advice and interpretation of results. DL provided expert advice on all issues and provided support throughout the study. All authors participated in editing the manuscript and read and approved the final version.

## Pre-publication history

The pre-publication history for this paper can be accessed here:

http://www.biomedcentral.com/1471-2458/10/757/prepub

## Supplementary Material

Additional file 1 **Frequency of births admitted at least once for ALRI before age 2 years by risk factor**. Click here for file
