# Supplementary material for: A retrospective population-based cohort study identifying target areas for prevention of acute lower respiratory infections in children
Source: BMC Public Health. 2010 Dec 7;10:757. doi: 10.1186/1471-2458-10-757 (PMC3004840; doi:10.1186/1471-2458-10-757)
Supplement: Additional file 1 — Frequency of births admitted at least once for ALRI before age 2 years by risk factor. [file 1471-2458-10-757-S1.PDF]

Additional file 1: Frequency of births admitted at least once for ALRI before age 2 years by risk factor

Risk factor	Aboriginal							Non-Aboriginal						
	No. of births	No. (%) admitted						No. of births	No. (%) admitted					
		Pneumonia		Bronchiolitis		Influenza			Pneumonia		Bronchiolitis		Influenza	
Gender (n=245,113)														
Male	8,889	990	(11.1)	1,416	(15.9)	109	(1.2)	116,575	2,722	(2.3)	4,683	(4.0)	613	(0.5)
Female	8,577	848	(9.9)	1,049	(12.2)	95	(1.1)	111,072	2,014	(1.8)	3,187	(2.9)	460	(0.4)
Gestational age (n=243,557)														
<33 weeks	479	92	(19.2)	147	(30.7)	14	(2.9)	2,284	177	(7.8)	340	(14.9)	46	(2.0)
33-34 weeks	411	58	(14.1)	95	(23.1)	12	(2.9)	2,591	94	(3.6)	190	(7.3)	23	(0.9)
35-36 weeks	1,213	163	(13.4)	231	(19.0)	22	(1.8)	9,347	260	(2.8)	592	(6.3)	74	(0.8)
≥37 weeks	14,855	1,458	(9.8)	1,906	(12.8)	151	(1.0)	212,377	4,186	(2.0)	6,711	(3.2)	926	(0.4)
Percent Optimal Birthweight (n=215,970)														
Low <85%	2,994	395	(13.2)	507	(16.9)	54	(1.8)	20,836	560	(2.7)	979	(4.7)	128	(0.6)
Normal 85-114%	10,038	1,047	(10.4)	1,354	(13.5)	116	(1.2)	160,577	3,364	(2.1)	5,373	(3.4)	763	(0.5)
High >=115%	1,103	108	(9.8)	137	(12.4)	12	(1.1)	20,422	435	(2.1)	698	(3.4)	94	(0.5)

Number of previous pregnancies (n=244,568)																	
0	3,957	365	(9.2)	501	(12.7)	52	(1.3)	67,316	1,268	(1.9)	1,425	(2.1)	269	(0.4)			
1	3,677	331	(9.0)	458	(12.5)	34	(0.9)	71,710	1,418	(2.0)	2,500	(3.5)	327	(0.5)			
2	2,977	332	(11.1)	437	(14.7)	28	(0.9)	43,749	972	(2.2)	1,764	(4.0)	206	(0.5)			
≥3	6,757	808	(12.0)	1064	(15.8)	90	(1.3)	44,425	1,073	(2.4)	2,175	(4.9)	270	(0.6)			
Season of birth (n=245,113)																	
Summer (Dec-Feb)	4,356	452	(10.4)	633	(14.5)	40	(0.9)	55,387	1,105	(2.0)	1,762	(3.2)	247	(0.5)			
Autumn (Mar-May)	4,606	539	(11.7)	729	(15.8)	54	(1.2)	58,161	1,339	(2.3)	2,727	(4.7)	292	(0.5)			
Winter (Jun-Aug)	4,371	465	(10.6)	642	(14.7)	62	(1.4)	56,820	1,175	(2.1)	2,116	(3.7)	290	(0.5)			
Spring (Sept-Nov)	4,133	382	(9.2)	461	(11.2)	48	(1.2)	57,279	1,117	(2.0)	1,265	(2.2)	244	(0.4)			
Mode of delivery (n=244,563)																	
Vaginal	12,862	1,401	(10.9)	1,816	(14.1)	153	(1.2)	134,660	2,843	(2.1)	4,752	(3.5)	618	(0.5)			
Instrumental	1,163	104	(8.9)	139	(12.0)	11	(0.9)	31,063	543	(1.7)	754	(2.4)	135	(0.4)			
Elective caesarean	1,328	123	(9.3)	199	(15.0)	14	(1.1)	35,119	685	(2.0)	1,355	(3.9)	173	(0.5)			
Emergency caesarean	2,014	208	(10.3)	306	(15.2)	26	(1.3)	26,354	660	(2.5)	1,003	(3.8)	146	(0.6)			
Maternal smoking during pregnancy (n=202,681)[a]																	

Yes	7,028	735	(10.5)	1,107	(15.8)	84	(1.2)	34,009	869	(2.6)	1,917	(5.6)	189	(0.6)
No	7,537	655	(8.7)	932	(12.4)	65	(0.9)	154,107	2,706	(1.8)	4,602	(3.0)	632	(0.4)
Maternal asthma during pregnancy (n=244,568) [a]														
Yes	1,485	125	(8.4)	242	(16.3)	16	(1.1)	19,338	486	(2.5)	1,039	(5.4)	119	(0.6)
No	15,883	1,711	(10.8)	2,218	(14.0)	188	(1.2)	207,862	4,245	(2.0)	6,825	(3.3)	953	(0.5)
Maternal age (years) (n=245, 038)														
<20	4,062	432	(10.6)	604	(14.9)	59	(1.5)	10,045	283	(2.8)	526	(5.2)	69	(0.7)
20-24	5,603	580	(10.4)	793	(14.2)	56	(1.0)	36,020	917	(2.5)	1,656	(4.6)	217	(0.6)
25-29	4,316	470	(10.9)	636	(14.7)	46	(1.1)	69,253	1,530	(2.2)	2,470	(3.6)	326	(0.5)
30-34	2,420	250	(10.3)	301	(12.4)	30	(1.2)	73,240	1,342	(1.8)	2,172	(3.0)	308	(0.4)
≥35	1,065	106	(10.0)	131	(12.3)	13	(1.2)	39,014	661	(1.7)	1,046	(2.7)	153	(0.4)
SEIFA Index of Disadvantage (n=218,124)														
0-10% (most disadvantaged)	5,740	689	(12.0)	856	(14.9)	66	(1.1)	17,398	486	(2.8)	885	(5.1)	108	(0.6)
11-25%	3,860	346	(9.0)	533	(13.8)	54	(1.4)	34,349	819	(2.4)	1,497	(4.4)	170	(0.5)
26-75%	4,144	363	(8.8)	509	(12.3)	48	(1.2)	105,349	2,133	(2.0)	3,477	(3.3)	513	(0.5)

76-90%	324	13	(4.0)	33	(10.2)	3	(0.9)	30,426	515	(1.7)	845	(2.8)	121	(0.4)
91-100% (least disadvantaged)	61	7	(11.5)	4	(6.6)	0	(0.0)	16,473	253	(1.5)	348	(2.1)	70	(0.4)
Accessibility/Remoteness Index of Australia (n=219,211)														
Major cities	5,545	374	(6.7)	704	(12.7)	107	(1.9)	153,134	2,786	(1.8)	5,096	(3.3)	828	(0.5)
Inner regional	1,058	74	(7.0)	128	(12.1)	9	(0.9)	22,100	536	(2.4)	752	(3.4)	61	(0.3)
Outer regional	2,381	276	(11.6)	337	(14.2)	26	(1.1)	18,849	617	(3.3)	877	(4.7)	63	(0.3)
Remote	2,500	244	(9.8)	339	(13.6)	13	(0.5)	8,406	238	(2.8)	297	(3.5)	26	(0.3)
Very remote	3,080	512	(16.6)	501	(16.3)	19	(0.6)	2,158	31	(1.4)	55	(2.6)	7	(0.3)

[a] Data on maternal smoking and maternal asthma only available from 1997-2005
